# Cerebellar and Occipital Alterations in Brain Perfusion in a Patient With Post-acute COVID-19 Encephalopathy Misdiagnosed As Primary Psychotic Disorder

**DOI:** 10.7759/cureus.52953

**Published:** 2024-01-25

**Authors:** Yuki Ikemizu, Yasunori Oda, Yuki Hirose, Tsuyoshi Sasaki, Masaomi Iyo

**Affiliations:** 1 Department of Psychiatry, Graduate School of Medicine, Chiba University, Chiba, JPN; 2 Research Center for Child Mental Development, Chiba University, Chiba, JPN; 3 Department of Child Psychiatry, Chiba University Hospital, Chiba, JPN

**Keywords:** 18f-fluorodeoxyglucose positron emission tomography (18f-fdg pet), single photon emission tomography (spect), encephalopathy, post-covid-19, post-acute covid-19, covid-19

## Abstract

We describe the case of an unvaccinated 21-year-old Japanese male who experienced psychotic symptoms attributed to encephalopathy, known as post-acute COVID-19 syndrome (PACS). One week after his discharge following the remission of a SARS-CoV-2 infection, he experienced hyperactive delirium and unexpected movements of his limbs. As COVID-19-associated encephalopathy was suspected as a cause of the psychotic symptoms, he was admitted to the Department of Neurology. He received antiviral and steroid pulse therapy, but his psychiatric symptoms did not improve completely. Consequently, he was admitted to our psychiatric ward with a diagnosis of a primary psychotic disorder. Although he did not take psychopharmacotherapy, he gradually achieved a remission of psychiatric symptoms. At three months post-SARS-CoV-2 infection, single-photon emission computed tomography (SPECT) revealed hypoperfusion in the bilateral cerebellar dentate nuclei and occipital lobes. However, no abnormal findings were observed on fluorine-18 fluoro-deoxy-glucose positron emission tomography (^18^F-FDG PET) at six months after the infection. This case indicates that (1) brain perfusion SPECT can be effective for detecting functional alterations in post-acute COVID-19-associated encephalopathy, and (2) it is necessary to carefully monitor patients' progress instead of quickly diagnosing a primary psychotic disorder.

## Introduction

The COVID-19 pandemic, caused by SARS-CoV-2, has affected the world since the end of 2019. According to the World Health Organization, there have been approximately 770 million COVID-19 cases globally. Although most people infected with SARS-CoV-2 get better without sequelae, some experience post-acute COVID-19 or post-COVID-19 symptoms. The terms *post-acute COVID-19* and *post-COVID-19* refer to the prolonged symptoms, which may be new or returning symptoms that individuals develop after their recovery from the initial, acute SARS-CoV-2 infection [1]. While COVID-19 targets mainly the body's respiratory organs, accumulating evidence suggests that COVID-19 can cause neurological disorders. The course of COVID-19 also appears to vary significantly among individuals, as do the symptoms of this disease.

There is growing concern about neuropsychiatric complications following SARS-CoV-2 infection. Almqvist et al. have drawn clinicians' attention to at least five classes of neurological complications: (1) cerebrovascular disorders, including ischemic stroke and macro/micro-hemorrhages; (2) encephalopathies; (3) para-/post-infectious immune-mediated complications, such as Guillain-Barre syndrome and acute disseminated encephalomyelitis (ADEM); (4) (meningo-)encephalitis, potentially with concomitant seizures; and (5) neuropsychiatric complications, such as psychosis and mood disorders [2]. Indeed, it was reported that one-third to two-thirds of patients with COVID-19 have been diagnosed with some type of neuropsychiatric disorder [3].

Encephalopathy is a broad term that encompasses a wide range of presentations and etiologies. The exact definition of the encephalopathy associated with COVID-19 has not been clarified [4]. Molecular imaging techniques such as brain fluorine-18 fluoro-deoxy-glucose positron emission tomography (^18^F-FDG PET) and single-photon emission computed tomography (SPECT) have been used for the diagnostic workup of neurological COVID-19 manifestations [5], and several ^18^F-FDG PET studies have revealed hypometabolism in various brain regions in patients experiencing post-COVID-19 symptoms [6]. Other studies used FDG-PET to investigate the longitudinal metabolic patterns in COVID-19 encephalopathy [7,8]. Both PET and SPECT are established methods for the differential diagnoses of neurodegenerative central nervous system disorders, and evidence about the usefulness of PET for COVID-19 continues to accumulate. A few studies using SPECT for COVID-19 have been conducted. However, these studies were restricted to 123I-FPCIT-SPECT to evaluate parkinsonism [9,10], and only a few researchers have employed N-isopropyl-p-(123I)-iodoamphetamine single-photon emission computed tomography (123I-IMP-SPECT) to assess brain changes associated with COVID-19 encephalopathy [11].

We describe the case of an individual who presented with psychosis due to encephalopathy even after the remission of his COVID-19 state. Since there were no abnormal findings on various tests, including magnetic resonance imaging (MRI) and an examination of the patient's cerebrospinal fluid (CSF), he was diagnosed with primary psychotic disorder and admitted to our psychiatric ward. However, 123I-IMP-SPECT was able to detect brain alterations in the patient, resulting in the diagnosis of post-acute COVID-19 encephalopathy.

## Case presentation

The patient was an unvaccinated 21-year-old Japanese male with no previous psychiatric history. In August 2021, he visited an emergency department due to the fever he was experiencing. Since his COVID-19 rapid antigen test result was positive (first day), he self-quarantined at a hotel for several days. However, he developed respiratory failure and was admitted to a COVID-19 response hospital (eighth day). He was immediately treated with minimal oxygen therapy, steroids, and remdesivir. He also received oral 6 mg dexamethasone for nine days. He was discharged without sequelae seven days after admission (15th day).

A week after his discharge, he gradually developed insomnia and anxiety and then began to experience unexpected chorea-like movements of his limbs. On the 25th day, he developed severe confusion. Since he also had a moderate fever, he was transferred to another COVID-19 response hospital by ambulance. At that hospital, the result of a rapid antigen test was negative, but the patient exhibited psychomotor excitation and ran screaming out of the hospital. He was finally restrained by police.

On the 26th day, he was brought to our hospital's emergency department. Neurological examinations revealed an altered level of consciousness, disorientation, and torsion dystonia. He was then considered to be in a delirious state. As COVID-19 associated with encephalitis was suspected, he was admitted to our hospital's neurology department. The result of a reverse transcription-polymerase chain reaction (RT-PCR) test for SARS-CoV-2 at that time was also negative. Blood screening results at his admission showed an increased white blood cell count (11.2 L × 10^3^/L) and an increased C-reactive protein (CRP) (3.37 mg/dL) level; the results of a CSF examination and the brain MRI consisting of T1, T2, FLAIR (fluid-attenuated inversion recovery), and DWI (diffusion-weighted imaging) were unremarkable.

From the day of the patient's admission, he was treated empirically with intravenous acyclovir for possible herpetic encephalitis. On the 29th day, he was still in a state of agitation and disorientation. He was thus administered haloperidol 10 mg/day, but no remarkable changes occurred. At this point, the patient presented a cranial nerve disorder resembling dysarthria and dysfunction of skilled motor activities. Further investigations, including CSF-PCR, electroencephalography (EEG), and an anti-NMDA (N-methyl-D-aspartate) receptor antibody test were performed, and all test results were within normal limits. However, the patient's clinical state was highly suggestive of encephalitis associated with COVID, and we thus administered steroid pulse therapy with intravenous 1,000 mg methylprednisolone for three days and then intravenous 60 mg methylprednisolone for three days.

After this corticosteroid therapy, the patient appeared to show significant improvement in his consciousness level and confusion. Based on the absence of characteristic findings, neurologists concluded that his symptoms were of primary psychotic disorder. However, he still experienced executive dysfunction and significant attentional deficits. He also presented verbal perseveration and rapid mood swings. On the 38th day, we decided to commit him to our psychiatric ward. As he gradually experienced an improvement of psychiatric symptoms, he was closely monitored without the use of psychotropic medication.

After his admission to the psychiatric ward, the patient achieved a remission of psychiatric symptoms. However, the impairment of the movement of his upper and lower limbs and the verbal fluency and dysarthria persisted. His results on the Clinical Assessment for Attention Test (CAT) consisting of a Visual Cancellation Task (VCT), a Symbol Digit Modalities Test (SDMT), and a Memory Updating Test (MUT) revealed that he also suffered significant attentional deficits (Table 1). We thus administered an intensive rehabilitation treatment consisting of physiotherapy and occupational therapy. 

**Table 1:** The results of CAT. The patient's performance on CAT is presented as raw scores and equivalent years.

Clinical Assessment for Attention Test (CAT)	Score	Equivalent years
Visual Cancellation Task (VCT)		
[Shape 1] Time	48.2 seconds	50s
Correct answers	98.2%	
Hit rate	100%	
[Shape 2] Time	48.4 seconds	50s
Correct answers	100%	
Hit rate	100%	
[Number] Time	74.5 seconds	50s
Correct answers	99.1%	
Hit rate	100%	
[Pseudonym] Time	83.5 seconds	30s
Correct answers	100%	
Hit rate	100%	
Symbol Digit Modalities Test (SDMT)	44.5%	60s
Memory Updating Test (MUT)		
Three digits	87.5%	40s
Four digits	75%	40s

**Figure 1:**
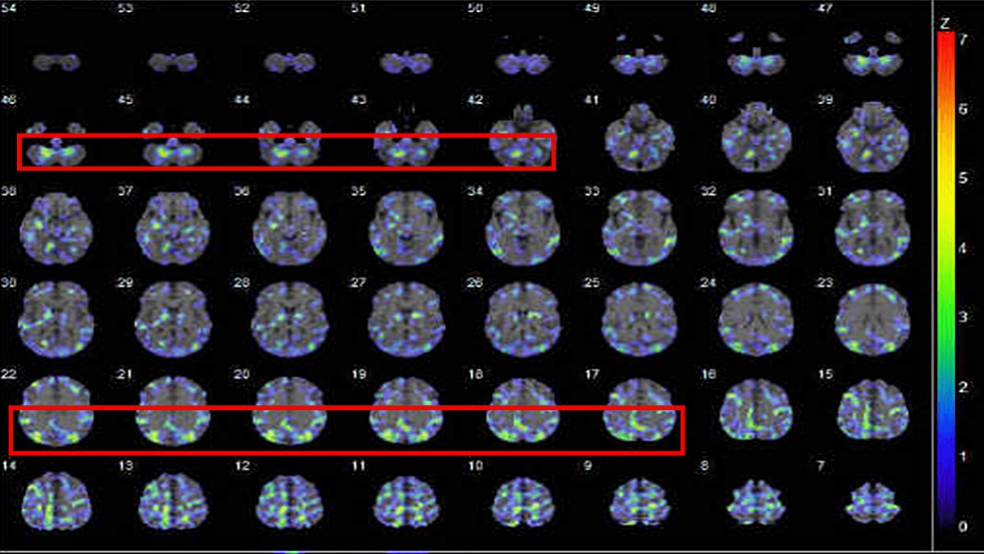
Brain N-isopropyl-p-(123I)-iodoamphetamine (IMP) single-photon emission computed tomography (SPECT) findings. The 123I-IMP-SPECT examination performed three months after the onset of the patient's SARS-CoV-2 infection indicated hypoperfusion in the bilateral cerebellar dentate nuclei and occipital lobes. The areas of hypoperfusion are shown on three-dimensional surface projection (3D-SSP: decrease).

After three weeks of rehabilitation at our hospital, the patient went to another hospital to continue rehabilitation. When he left our hospital, his Global Assessment of Functioning (GAF) score had improved from 10 points to 65 points and his score on the Brief Psychiatric Rating Scale (BPRS) had improved from 81 points to 13 points. After his discharge from the rehabilitation hospital, he visited our hospital regularly. A SPECT examination that was conducted three months after the onset of the patient's SARS-CoV-2 infection revealed hypoperfusion in the bilateral cerebellar dentate nuclei and occipital lobes (Figure 1). However, at six months after the onset of the patient's infection, there were no abnormal findings on ^18^F-FDG PET. Nine months after the onset of the patient's SARS-CoV-2 infection, he continued to exhibit a mild attention deficit and impaired fine motor skills in both arms. 

## Discussion

We have presented the case of an adult with psychosis associated with encephalopathy following the remission of COVID. Although this patient's case would be classified as PACS, he still experienced an attention deficit and impaired fine motor skills in both arms nine months after the onset of his SARS-CoV-2 infection. Since the patient's results on several tests, including a CSF examination and MRI, were unremarkable, he was admitted to our psychiatric ward for possible primary psychotic disorder. The eventual use of 123I-IMP-SPECT finally detected hypoperfusion in the patient's bilateral cerebellar dentate nuclei and occipital lobes. He was thus able to discontinue his psychiatric visits and function sufficiently without antipsychotic medication.

While several ^18^F-FDG-PET imaging studies of post-acute COVID-associated encephalopathy have been reported, accumulating evidence suggests that FDG-PET hypometabolism and cognitive impairment are associated in patients with a COVID-19 infection after a maximum of one-year post-onset [12]. In our patient's case, ^18^F-FDG PET did not show abnormal findings at six months post-onset. There are a few case reports of encephalopathy with brain perfusion abnormalities observed on 123I-IMP-SPECT. Interestingly, in our patient's case, 123I-IMP-SPECT revealed hypoperfusion in the bilateral cerebellar dentate nuclei and occipital lobes. The dentate nucleus is the largest cerebellar nucleus, and it is involved in the fine control of voluntary movement, cognition, language, and sensory functions [13]. The human dentate nuclei are divided into default-mode, salience-motor, and visual processing territories [14]. The occipital lobe is the visual information processing area of the brain. It is also associated with visuospatial processing, distance and depth perception, color determination, object and face recognition, and memory formation [15]. We thus speculate that the hypoperfusion observed in our patient's cerebellar dentate nuclei and occipital lobes might have been involved in his impaired fine motor skills and decline in CAT scores, especially on the VCT. Since this patient's neurologic manifestations and psychological examination results are explainable by the hypoperfusion in the cerebellar dentate nuclei and occipital lobes, it is reasonable to diagnose him with post-acute COVID-associated encephalopathy. The present case highlights the merits of performing 123I-IMP-SPECT when difficulty in diagnosing patients with COVID-19 is encountered. Further investigations are needed to elucidate this issue.

The Global COVID-19 Neuro Research Coalition recently strongly recommended that delirium, subsyndromal delirium, and coma be used as criteria for suspecting COVID-19 encephalopathy [4]. However, the diagnosis of post-acute COVID-19 encephalopathy, in which the SARS-CoV-2 status is already negative, is very difficult even for neurologists. About the present patient's case, since psychiatric symptoms are rarely the primary symptom in ADEM, it is unlikely that this case was ADEM. In contrast, anti-NMDA receptor encephalitis is often marked by psychiatric symptoms, but the anti-NMDA receptor antibody was negative in our patient. Since he had predominantly psychiatric symptoms with no abnormal findings on the head MRI, it is highly unlikely that the other type of anti-neuronal cell surface antigen antibodies would be detected. The hospital's neurologists thus diagnosed our patient with primary psychotic disorder, not post-acute COVID-associated encephalopathy. However, 123I-IMP-SPECT revealed the patient's brain alterations, resulting in the diagnosis of post-acute COVID-19 encephalopathy. If he had been diagnosed with primary psychotic disorder without further testing, he may have been on antipsychotic medication for a long period. Given the difficulty in diagnosing encephalopathy with the usual tests, including CSF examination and brain MRI, it appears that clinicians need to prescribe antipsychotics for patients with possible encephalopathy after thorough consideration.

## Conclusions

In conclusion, we successfully treated a patient with post-acute COVID-19 encephalopathy without psychotropic medication. 123I-IMP-SPECT was able to visualize hypoperfusion in the patient's bilateral cerebellar dentate nuclei and occipital lobes. If there is even a slight possibility of post-acute COVID-19 encephalopathy, it is necessary to carefully monitor the patient's progress rather than easily diagnose primary psychotic disorder. It is also worthwhile to perform 123I-IMP-SPECT.
